# How Many People Living with HIV Will Be Additionally Eligible for Antiretroviral Treatment in Karnataka State, India as per the World Health Organization 2013 Guidelines?

**DOI:** 10.1371/journal.pone.0107136

**Published:** 2014-09-05

**Authors:** Sunil Kumar Dodderi, Ajay M. V. Kumar, Balaji R. Naik, Avinash Kanchar, Rewari B. B., Anthony D. Harries

**Affiliations:** 1 Karnataka State AIDS Prevention Society, Bangalore, India; 2 National AIDS Control Organization, Ministry of Health and Family Welfare, Government of India, New Delhi, India; 3 International Union Against Tuberculosis and Lung Disease, South-East Asia Office, New Delhi, India; 4 World Health Organization country office for India, New Delhi, India; 5 World Health Organization, Geneva, Switzerland; 6 International Union Against Tuberculosis and Lung Disease, Paris, France; 7 Department of Infectious and Tropical Diseases, London School of Hygiene and Tropical Medicine, London, United Kingdom; Curtin University, Australia

## Abstract

**Background:**

The National AIDS control programme (NACP) in India is currently following the World Health Organization (WHO) 2010 antiretroviral therapy (ART) guidelines. In 2013, the WHO revised its recommendations for initiating ART among people living with HIV (PLHIV) by increasing the threshold for ART initiation to a CD4 count ≤500 cells/uL. For certain patient groups, ART is recommended irrespective of CD4 count (PLHIV with active tuberculosis, hepatitis B virus infection, pregnant and breast feeding women, children aged under five years and those living in a sero-discordant relationship). In this operational research, we assess the effect of applying this recommendation on the number of PLHIV additionally eligible for ART.

**Methods:**

This was a cross-sectional analysis of routinely collected programme data from all PLHIV registered in Karnataka State (population 60 million), India in 2012.

**Results:**

Of 37,044 PLHIV, 27,074 (73%) were eligible for initiating ART as per WHO-2010 criteria. As per the WHO-2013 criteria (CD4 count ≤500 and all pregnant women and under-five children irrespective of CD4 count), an additional 5104 (14%) HIV-infected people would be eligible for initiating ART. There were no data to inform the additional patient load due to sero-discordance.

**Conclusion:**

Adopting the WHO-2013 guidelines for India has important resource implications. However, given the significant patient and programmatic benefits of adopting the new guidelines, this has been considered favourably by the NACP in India and steps are being planned to integrate ART care into the general health system to cope with the increased numbers of patients.

## Introduction

With the progress in the scale-up and access to free antiretroviral therapy (ART) across the globe, Human Immunodeficiency Virus (HIV) infection, has now become a chronic manageable illness rather than a virtual death sentence a decade ago [1]. HIV incidence and mortality are declining steadily [1]. However, with an estimated 34 million people living with HIV (PLHIV), 2.4 million new HIV infections and 1.7 million deaths in 2011, HIV continues to be the most common infectious cause of mortality in the world, with sub-Saharan Africa being its epicentre [1]. India is considered a country with a concentrated HIV epidemic and contributes to about 10% of the global burden in absolute terms [1]. The HIV epidemic in India is showing a declining trend and in 2011, about 2.1 million people were living with HIV in India with an estimated 0.12 million new infections and 0.15 million deaths [2].

ART is the most important life-saving intervention for PLHIV. HIV is probably the only infectious disease where there is a waiting period before treatment is initiated. This has been for various reasons that include the toxic nature of the ARV drugs, concerns about non-adherence and risk of drug resistance if treatment is started too early and the need to weigh up benefits and harms of starting treatment at certain CD4 count thresholds. The first guidance from WHO in 2002 recommended ART for those in WHO clinical stage 3 and 4 and those with a CD4 lymphocyte count of ≤200 cells/uL. With availability of safer and inexpensive treatment options and evidence of benefit of earlier ART in terms of reduced morbidity and mortality, WHO raised the CD4 cut-off for starting ART to ≤350 cells/uL in 2010. In the first ever consolidated guidelines on the use of antiretroviral drugs published in 2013, the WHO recommends to further raise the threshold for ART initiation to a CD4 count ≤500 cells/uL in adults, adolescents and children aged five years and above. For certain patient groups, ART is recommended irrespective of CD4 count e.g. PLHIV having active TB disease, hepatitis B virus infection with severe chronic liver disease, pregnant and breast feeding women, children aged under five years, and those living in a sero-discordant relationship to reduce HIV transmission to uninfected partners (**Table 1**) [3]. This decision comes in the wake of recent and growing evidence of the benefits of starting ART early, with the potential for reducing HIV-related morbidity and mortality and preventing onward transmission of HIV. Thus, early initiation of ART has both individual clinical benefits and population level benefits.

**Table 1:** Comparison of WHO guidelines for ART initiation among people living with HIV in the year 2010 and 2013.

Population	Target Population	2010 ART guidelines	2013 ART guidelines
Adults and Adolescents	HIV infected individuals	CD4 count <350 cells/mm^3^ or WHO clinical stage 3 or 4 regardless of CD4 cell count	**CD4 count ≤500 cells/mm^3^** or WHO clinical stage 3 or 4 regardless of CD4 cell count
Adults and Adolescents	HIV infected pregnant and breastfeeding women	CD4 count ≤350 cells/mm^3^ regardless of clinical symptoms or WHO clinical stage 3 or 4 regardless of CD4 cell count	**Regardless of CD4 cell count or WHO clinical stage**
Adults and Adolescents	HIV infected partners in serodiscordant couple relationship(s)	No recommendation established	**Regardless of CD4 cell count or WHO clinical stage**
Adults and Adolescents	HIV/TB co-infection	Presence of active TB disease, regardless of CD4 cell count	**No change**
Adults and Adolescents	HIV/HBV co-infection	Evidence of chronic active HBV disease, regardless of CD4 cell count	**Evidence of chronic HBV disease with advanced stage liver disease (e.g. cirrhosis), regardless of CD4 cell count**
Children	HIV infected children ≥5 years old	CD4 **≤**350 cells/mm^3^ or WHO clinical stage 3 or 4 regardless of CD4 cell count	**CD4 count ≤500 cells/mm^3^** or WHO clinical stage 3 or 4 regardless of CD4 cell count
Children	HIV infected children 1–5 years old	1. Between 12 and 24 months of age, regardless of CD4 count or WHO clinical stage. 2. Between 24 and 59 months of age with CD4 count of ≤750 cells/mm3 or CD4% ≤25, or whichever is lower, regardless of WHO clinical stage.	**Regardless of CD4 cell count and clinical stage**
Children	HIV infected infants <1 year old	All infants, regardless of CD4 cell count and clinical stage	**No change**

WHO-World Health Organization; ART-antiretroviral therapy; HIV-Human immunodeficiency virus; TB – Tuberculosis; HBV – Hepatitis B Virus.

The National AIDS control programme (NACP) in India is currently following the WHO 2010 ART guidelines [4]. Adoption of the new WHO recommendations is likely to increase the number of PLHIV eligible for ART in India and there are concerns that it may pose an increased burden on the NACP as well as the general health system. However, currently there is no information on the number and proportion of PLHIV that will be additionally eligible for ART if the NACP decides to adopt 2013 WHO guidelines. This information is critical for programme planning including forecasting of drug requirements, procurement and supply chain management of new formulations of drugs and possible changes in delivery of ART services within health systems to cope with increased numbers of people receiving ART. In this operational research, we therefore aimed to assess the number of PLHIV who were additionally eligible for ART in a large south Indian state of Karnataka. The specific objective was to determine among a cohort of PLHIV registered for HIV care in the year 2012 in Karnataka, the number (proportion) eligible for ART if the WHO 2013 ART guidelines were followed.

## Methods

### Ethics considerations

Ethics approval was obtained by the Ethics Advisory Group of International Union Against Tuberculosis and Lung Disease, Paris, France. Administrative approval to conduct the study was obtained from the National AIDS Control Organization in India. Since this was a retrospective review of existing records and did not involve any direct patient interaction, ethics committee waived the need for individual informed consent.

### Study Design

This was a cross-sectional study involving secondary analysis of data routinely recorded under the National AIDS Control Programme (NACP).

### Setting

Karnataka, with 30 districts and a population of 61 million, is one of four large states in South India facing a relatively advanced HIV epidemic, with the adult HIV prevalence in some districts exceeding 1%. According to national estimates in 2012, Karnataka state had a HIV-prevalence of 0.52% with 0.21 million persons living with HIV [5].

There were 565 stand-alone HIV testing facilities, 1050 facility integrated HIV testing facilities and 49 ART centers in the State. The primary aim of the HIV testing facilities is to provide information, counselling and HIV testing services. All HIV positive persons diagnosed at testing centres are referred to the nearest ART centre for further management. HIV positive patients, who reach ART centres are registered for HIV care, are assessed clinically as per WHO clinical staging including CD4 count assessments and if found eligible for ART according to national guidelines, they are initiated on ART [6], [7]. Most of the ART centres are situated in tertiary care facilities and all supportive patient care, like investigations or facilities for hospitalization, is integrated within the general health system. India currently follows WHO 2010 ART guidelines. All services including diagnosis of HIV, CD4 count assessments and ART are provided free of cost for the patients.

### Study population and Study period

All PLHIV newly diagnosed and registered for HIV care at ART centres in Karnataka State in the year 2012 constituted the study population. The study was conducted during the year 2013.

### Data collection procedure and data variables

The data variables included pre-ART number, age, sex, WHO clinical staging, CD4 lymphocyte count at the time of registration, co-existing TB disease (Yes/No) and whether pregnant (Yes/No) at the time of registration. These variables were extracted from the electronic medical record maintained at the ART centres during the month of August 2013. Original data sources for this record included the pre-ART patient register and patient treatment cards maintained at each centre.

### Data entry and analysis

Since the data were already present in the electronic format, double data entry and validation was not considered. Abstracted data from the Microsoft Excel database were imported into EpiData software and analysed (Version 2.2.2.182, EpiData Association, Odense, Denmark).

## Results

Of 49 ART centres in the State, data were available from 47 centres. Two ART centres which operated under a public-private mix model and accounted for less than 0.5% of all PLHIV registered in 2012 were not included due to lack of data. There were 37,307 HIV-positive patients registered for HIV care (pre-ART) in these centres. Basic demographic and clinical characteristics of the study population are described in **Table 2**. Of all HIV-infected people, 50% were males and the median (IQR) age was 35 (28–42) years. About 6% were children (aged less than 15 years) and about 2% were aged less than five years. About 13% had TB and 4% were pregnant at enrolment. The median (IQR) CD4 count was 242 (122–415) and was significantly higher in females [275(144–466)] as compared with males [208(106–361)]. About 75% were assessed to be in WHO clinical stage 1 or 2.

**Table 2:** Demographic and clinical characteristics of HIV infected people registered for pre-ART care in Karnataka State, India, 2012 (N = 37307).

Characteristics	Number	Percentage
**Sex**		
Male	18662	50.0
Female	18520	49.7
Transgender	85	0.2
Not recorded	40	0.1
**Age in years**		
0–4	617	1.7
5–14	1560	4.2
15–24	3047	8.2
25–34	11284	30.2
35–44	12425	33.3
45–54	5776	15.5
55–64	1985	5.3
65 and above	575	1.5
Not recorded	38	0.1
**Tuberculosis**	4745	12.7
**Pregnant women**	1494	4.0
**WHO Clinical staging**		
Stage 1	12677	34.0
Stage 2	15645	41.9
Stage 3	6642	17.8
Stage 4	1793	4.8
Unknown	550	1.5
**CD4 Count**		
≤50	3163	8.5
51–250	16072	43.1
251–350	5955	16.0
351–500	5219	14.0
501 and above	6491	17.4
Unknown	407	1.1

WHO-World Health Organization; ART-antiretroviral therapy; HIV-Human immunodeficiency virus;

The number and proportion eligible for initiating ART as per WHO-2010 guidelines and 2013 guidelines are compared in **Table 3**. Of all PLHIV registered, eligibility could be assessed for 37044 (99%). As per the WHO-2010 criteria, 27074 (73%) were eligible for initiating ART and nearly 85% of those eligible were initiated on ART. As per the WHO-2013 criteria, an additional 5104 (14%) HIV-infected people would be eligible for initiating ART if all recommendations on CD4 cell count, pregnant women and children under-five years were followed, resulting in 87% in total being eligible for treatment.

**Table 3:** Number of HIV-infected people eligible for ART as per the current and new WHO criteria for ART initiation, Karnataka State, India, 2012 (N = 37044*).

Eligibility Criteria	Number eligible	Percentage	Number (%) additionally eligible
**WHO-2010 ART Guidelines**	27074	73.1	
**WHO-2013 ART Guidelines (CD4 count ≤500)**	31483	85.0	4409 (11.9)
**WHO-2013 ART Guidelines (all pregnant women irrespective of CD4 count)**	27981	75.5	907 (2.4)
**WHO-2013 ART Guidelines (all children under five years irrespective of CD4 count)**	27262	73.6	188 (0.5)
**WHO-2013 ART Guidelines (CD4 count ≤500+all pregnant women+ all under-five children irrespective of CD4 count)**	32178	86.9	5104 (13.8)

WHO-World Health Organization; ART-antiretroviral therapy; HIV-Human immunodeficiency virus;

*Eligibility could not be assessed for 263 patients.

## Discussion

This is the first study from India assessing the potential resource implications of adopting the WHO 2013 ART guidelines. Evidence from this large cohort of PLHIV from Karnataka state provides programmatically useful information from a planning perspective. Several points require further comment.

First, we found that nearly 90% of PLHIV would be eligible for ART as per the new recommendations, an addition of about 15% when compared to WHO 2010 recommendations. Adopting the new strategy will require increases in costs, manpower, infrastructure and drug requirements and the logistics need to be carefully planned to ensure smooth implementation of services [8]. In addition to clinical and immunological benefits, early ART has operational benefits too. Previous studies have shown better patient retention among those who are ‘on ART’ as compared to those who in ‘Pre-ART care’ [9]. So, adopting the new WHO guidelines will be of benefit for both the patients and the programme. Given the preparedness of the well-resourced NACP in India, this should be feasible and a decision to adopt the new WHO guidelines has been announced by the Union Health and Family Welfare Minister in a recent press release [5].

Second, we could not assess how many PLHIV would be eligible for ART if the criterion of sero-discordance was applied as we did not capture this data on this aspect. Although recommended by the NACP in India, information on HIV status of partners was not systematically documented in the treatment cards. We could not assess if it was merely due to poor documentation or suboptimal uptake of HIV testing among the partners. Previous studies analysing nationally representative data from the National Family Health Survey – 3 have indicated that among married couples with a HIV-infected partner; nearly 75% were sero-discordant [10], [11]. If we apply this figure to our cohort, then nearly all PLHIV would be eligible for ART. From a public health perspective, this would mean ‘immediate and universal ART’ for every person with HIV and is in alignment with the vision of NACP phase-IV for the period 2012–17 [5].

Third, implementation of the new strategy would mean that many PLHIV would be started on ART, while they are still asymptomatic and at higher CD4 counts. To avoid side-effects and improve treatment adherence over the long term in such patients, there will be a need to use tenofovir more frequently in the first line regimens, as recommended in the 2013 WHO Guidelines, which may further add to the costs.

Fourth, the current vertical structure of the programme (with delivery of services through a centralized network of ART centres with dedicated staff) may make it difficult to cope with the increased demand for services that will arise due to the new policy. While there have been some efforts by NACP in India to create a network of ‘Link ART centres’ at sub-district level health facilities to increase the access for PLHIV, this may not be sufficient. Hence, innovative strategies for delivery of ART may be required, such as further decentralisation of services, better integration and linkage of services and task shifting of personnel, all of which are broadly recommended by WHO. This will help in mitigating the costs and is in line with the overall vision of the Government of India to integrate NACP into the general health system [5].

There were a few limitations to our study. First, we did not conduct a costing exercise to assess the cost implications of adopting the new strategy. A full economic assessment would have added value to our findings but was beyond the scope of the current effort. This is a major limitation and needs to be addressed in future research. Second, this was an operational research and relied on the routinely recorded data with its inherent limitations. Third, we did not find data on HIV status of the partner and HIV/Hepatitis B co-infection documented in the treatment cards. Having this information would have helped in more accurate estimation of ART eligibility. Fourth, while the study used a large cohort, it came from one state in South India and cannot be claimed to be nationally representative. So, similar analyses should be conducted from other states or in a nationally representative sample before the findings can be generalized.

In conclusion, transitioning to the WHO 2013 ART guidelines in India would virtually mean ‘immediate and universal ART’ for all PLHIV with substantial resource implications. However, given the significant patient and programmatic benefits of adopting the new guidelines, this has been considered favourably by the NACP in India and steps are being planned to integrate ART care into the general health system to cope with the increased numbers of patients.
